# Genetic Variation in Circadian Rhythm Genes CLOCK and ARNTL as Risk Factor for Male Infertility

**DOI:** 10.1371/journal.pone.0059220

**Published:** 2013-03-18

**Authors:** Alenka Hodžić, Momčilo Ristanović, Branko Zorn, Cane Tulić, Aleš Maver, Ivana Novaković, Borut Peterlin

**Affiliations:** 1 Institute of Medical Genetics and Department of Obstetrics and Gynecology, University Medical Centre Ljubljana, Slovenia; 2 Institute of Human Genetics, Faculty of Medicine, University of Belgrade, Serbia; 3 Institute of Urology and Nephrology, Faculty of Medicine, University of Belgrade, Serbia; Instituto de Ciencia de Materiales de Madrid - Instituto de Biomedicina de Valencia, Spain

## Abstract

**Background:**

The circadian system has a major role in maintaining homeostasis and proper body functions including reproductive capacity. The aim of this study was to examine whether there is an association between genetic variability in the primary clock genes CLOCK and ARNTL and male infertility in humans.

**Methodology/Principal Findings:**

We performed a case-control study, where we searched for an association between polymorphisms of CLOCK and ARNTL genes and male infertility in 961 Slovenian and Serbian Caucasian men. The study group consisted of 517 patients with idiopathic infertility and a control group of 444 fertile men. A statistically significant difference was found in genotype distribution between the two groups in the CLOCK gene: rs11932595 (p = 6·10^−5^, q = 4·10^−4^, OR equaled 1.9 with 95% CI 1.4–2.7), rs6811520 (p = 2·10^−3^, q = 8·10^−3^, OR = 1.7 with 95% CI 1.2–2.2) and rs6850524 (p = 0.01, q = 0.02, OR = 1.4 with 95% CI 1.1–1.9). Further analyses of haplotypes were consistent with genotyping results.

**Conclusions/Significance:**

We provide evidence that genetic variability in the CLOCK gene might be associated with male infertility warranting further confirmation and mechanistic investigations.

## Introduction

Infertility affects about 9% couples [1] and male factors contribute to approximately half of them [2]. About one third of infertility cases are unexplained (idiopathic male infertility), mostly due to our poor understanding of basic molecular mechanisms underlying male fertility [3], [4]. It is estimated that genetic factors are implicated in the pathogenesis of 50% males with idiopathic male infertility [5].

There is a growing body of evidence that circadian rhythms have a major role in maintaining homeostasis and proper body function including reproductive capacity [6]. This is highlighted most obviously in mutant mouse models whereby mutations in the Bmal1 gene significantly reduce fertility in male mice [7]. The circadian clock system as well as primary clock genes, are highly conserved between species. It is known that the circadian system influences testosterone production in humans, showing morning peaks and low levels in the evening [8], [9], [10]. Moreover, serum levels of sex steroids have been associated with genetic variants in circadian rhythm genes [11].

Molecular components of the circadian clock network represent positive and negative transcriptional-translational feedback loops of many genes. CLOCK and ARNTL genes represent the central node in the network, generating a positive loop, and heterodimerizeing and initiating the transcription of other clock genes. Resultant proteins forming the negative feedback loop inhibit the CLOCK and ARNTL transcriptional activity [12].

Therefore, we hypothesized that genetic variability of the CLOCK and ARNTL genes may be associated with male infertility in humans. To test the hypothesis we performed a retrospective case-control genetic association study of polymorphic sites in these two genes on a population of patients with male infertility in comparison with fertile male control population.

## Materials and Methods

### Ethics Statement

The study was approved by committees in both countries participating in the study: Slovenian National medical ethics committee (reference number: 73/05/12) and by the Ethics committee at Medical faculty at University of Belgrade, Serbia (reference number: 29/II-3). All patients gave informed written consent to participate in the study.

### Subjects

Male partners of infertile couples, attending the infertility outpatient clinic of the Department of Obstetrics and Gynecology, University Medical Centre Ljubljana, and the Institute of Human Genetics, and Institute of Urology and Nephrology, Faculty of Medicine, University of Belgrade, participated in the study. The diagnosis of infertility was based on clinical assessment (testicular volume was measured using Prader's orchidometer), semen analysis, follicle stimulating hormone (FSH) testing and histologic evaluation of testicular biopsy specimens. Exclusion criteria were: patients with a history of testicular carcinoma, obstructive azoospermia including congenital bilateral absence of vas deferens, cytogenetic abnormalities and Y chromosome microdeletions.

Clinical, sperm and hormonal characteristics of the 517 infertile patients are summarized in Table 1.

**Table 1:** Clinical, sperm and hormonal characteristics of the 517 infertile men.

	Non obstructiveazoospermia(n = 219)	Oligoasthenoteratozoospermia (n = 298)			
		0.1–0.9×10^6^sperm/ml(n = 112)	≥1–4×10^6^sperm/ml(n = 45)	5–19×10^6^sperm/ml(n = 71)	≥20×10^6^sperm/ml(n = 34)
Testicular volume (right/left) (ml)	10.5±6.5/10.4±6.0	12.2±4.8/11.9±4.9	12.5±4.5/13.7±4.8	14.6±4.2/14.7±4.9	19.6±4.8/19.3±4.7
Sperm concentration(×10^6^ sperm/ml)	0	0.37±0.2	2.9±1.0	8.1±3.4	59.8±46.9
Rapid progressive motility(%)	0	4.2±1.7	10.1±8.1	12.6±10.0	26.3±12.9
Normal morphology (%)	0	5.1±7.1	8.2±8.9	10.3±9.2	24.9±14.4
SerumFSH level (IU/l)	20.9±16.6 (n = 201)	11.8±8.9 (n = 83)	9.2±5.0 (n = 36)	4.1±1.0 (n = 47)	2.2±0.9 (n = 2)

Values are given as means ± SD.

The control group of proven fertility consisted of 444 healthy males, who were fathers of at least one child and who reported no history of infertility. The seminal parameters and biopsy were not assessed for subjects in the control group. All patients and control subjects were Caucasian of the Slavic (Slovene or Serbian) origin and were recruited in a consecutive manner.

### Semen Parameters

In the 517 infertile men, semen analysis was performed according to the World Health organization (WHO) criteria [13]. Sperm was assessed in terms of volume, concentration, rapid progressive motility and normal morphology.

### Hormonal Parameters

Serum FSH was measured by Microparticle Enzyme Immunoassay (AxSYM System, Abbott Laboratories, Chicago, IL, USA); the reference interval for FSH was 1–8 mIU/ml.

### Testicular Biopsy and Histological Evaluation

Testicular biopsy and histological evaluation was performed in 77 patients with diagnosis of non obstructive azoospermia. In all patients testicular biopsy was performed for diagnostic and therapeutic (sperm recovery followed by cryostorage) purposes.

Following unilateral hemiscrototomy, a small incision was made and at least two samples of testicular tissue were taken from each testis. A systematic histological evaluation was performed under light microscopy; more than 100 seminiferous tubules were scored for each patient and results expressed as a relative number of tubules showing Sertoli cells, spermatogonia, spermatocytes, round and elongated spermatids, and spermatozoa. The diagnoses were as follows: Sertoli-cell only syndrome (SCOS) alone 46.8%, Maturation arrest (MA) alone 11.3%, Germinal hypoplasia (GH) alone 7.8%, and following combinations: GH+MA 14.1%, SCOS+MA 11.3%, GH+SCOS+MA 6.3% and GH+SCOS 2.1%.

### SNP Selection and Genotyping

Genetic variants in both key circadian rhythm-regulating genes, CLOCK and ARNTL, were genotyped in this study. Eight tagging single nucleotide polymorphisms (SNPs) were chosen from both genes. Of these, four SNPs, rs11932595, rs6811520, rs6850524, and rs13124436 were selected in the CLOCK gene, and four SNPs, rs3789327, rs1481892, rs4757144, and rs12363415 in the ARNTL gene. We based the selection of SNPs on the known genetic linkage in both genes, according to HapMap Phase 3 (http://www.hapmap.org). The SNPs were regarded as proxies for neighbouring SNPs, when their pairwise r^2^ values exceeded 0.80. The set of most representative tagSNPs for ARNTL and CLOCK gene regions was obtained using the Tagger algorithm available through the Haploview software (Haploview, version 4.2) [14]. To maximize statistical power, only the SNPs with minor allele frequency values exceeding 0.05 were included in the investigated set.

Genomic DNA was isolated from the peripheral blood samples using standard procedures. SNPs genotyping was carried out by real time PCR method performed on 7000 Sequence Detection System (Applied Biosystems, Foster City, CA, USA) using KASPar SNP genotyping chemistry. The PCR reaction mix of 8 µL final volume consisted of 4 µL of DNA sample, 4 µL of Reaction Mix 2X, 0.11 µL Assay Mix, 0.064 µL 50 mM MgCl2, and 3.826 µL H2O. The protocol for PCR amplification was: initial denaturation step at 94°C for 15 minutes, then 20 cycles of denaturation at 94°C for 10 sec, followed by 57°C or 61°C for 5 sec, 72°C for 10 sec, 94°C for 10 sec, 57°C or 61°C for 20 sec, and final extension at 72°C for 40 sec.

The allelic discrimination analysis was performed using SDS Software Version 1.2 (Applied Biosystems, Foster City, CA, USA).

Genotype assignment was performed and interpreted independently by two investigators.

### Statistical Analysis

Significances of associations between allelic and genotype frequencies and disease status were analyzed using the Chi-square test (χ2). Odds ratios (OR) and their respective 95% confidence intervals (CI) were also calculated to compare allelic and genotype distribution in patients and control subjects. Analyses were performed using R statistical language (R 2.15.0 for Windows). χ2 goodness-of-fit tests for deviation of genotype distribution from those predicted by Hardy-Weinberg equilibrium were also calculated, providing an additional quality control step of genotyping process. The investigated associations were regarded as significant when they reached p≤0.05.

As multiple SNPs were investigated, appropriate corrections of significance values were also applied using the Benjamini-Hochberg correction method (false-discovery rate – FDR values). We studied the extent of genetic linkage between SNPs using Haploview software version 4.2, which enables calculation of D’ and r2, representing the extent of pairwise linkage between two SNPs.

Haplotype frequencies and haplotype-disease associations were estimated using haplo.stats package for R (http://mayoresearch.mayo.edu/mayo/research/schaid_lab/software.cfm). Functions in haplo.stats package permit the calculation of indirectly measured haplotypes, under the founding assumption that subjects in the study are not related and the linkage phase unknown [15]. Here, the *haplo.score* function was used to directly ascertain differences in haplotype distributions across the groups of infertile men and control individuals. A global test of association, and per-haplotype association test were performed for both investigated genes. To reduce the effects of multiple testing we excluded all haplotypes with a frequency below 5% from downstream tests.

For power calculations, the *pbsize2* function belonging to the *gap* package for R was utilized, which allows accurate power estimations under a variety of disease inheritance models (available at web address: http://cran.r-project.org/web/packages/gap/index.html).

## Results

Genotype frequencies of investigated polymorphisms were in accordance with those predicted by the Hardy-Weinberg equilibrium in the study and in the control group (p<0.05), with the exception of rs12363415, which was excluded from further analyses. Genotype and allelic distribution of the CLOCK and ARNTL polymorphisms of the 517 infertile man and 444 fertile controls are shown in Table 2.

**Table 2:** Genotype and allelic distribution of the CLOCK and ARNTL polymorphisms of the 517 infertile men and 444 fertile controls.

		Alleles		Genotype counts (%)								
Gene	SNP	1	2	Infertile male			Controls			P value	χ^2^	Q value
				11	12	22	11	12	22			
CLOCK	rs11932595	G	A	129(26.16)	235(47.66)	129(26.16)	64 (15.45)	201 (48.55)	149(35.99)	6·10^−5^	19.25	4·10^−4^
CLOCK	rs6811520	T	C	66(14.76)	198(44.29)	183 (40.93)	59(15.60)	208(55.02)	111 (29.36)	2·10^−3^	12.59	8·10^−3^
CLOCK	rs6850524	G	C	182(35.82)	245(48.22)	81(15.94)	109(27.87)	195(49.87)	87(22.25)	0.01	9.14	0.02
CLOCK	rs13124436	G	A	225(46.10)	216(44.26)	49(9.63)	217(51.05)	170(40)	38(8.94)	0.299	2.41	0.39
ARNTL	rs3789327	T	C	148(29.6)	237(47.4)	115(23)	143(33.96)	202(47.98)	76(18.05)	0.129	4.09	0.20
ARNTL	rs1481892	G	C	228(46.43)	221(45.01)	42(8.55)	193(45.62)	200(47.28)	30(7.09)	0.637	0.90	0.72
ARNTL	rs4757144	G	A	107(21.79)	244(49.69)	140(28.51)	111(26.24)	176(41.60)	136(32.15)	0.047	6.12	0.09
ARNTL*	rs12363415	G	A	26(6.31)	112(27.18)	274(66.50)	29(6.90)	115(27.38)	276(65.71)	0.935	0.13	0.93

* ARNTL SNP rs12363415 showed a departure from the Hardy-Weinberg equilibrium and was excluded.

We found a statistically significant difference in the allelic distribution of rs11932595 (p = 6·10^−5^, q = 4·10^−4^), rs6811520 (p = 2·10^−3^, q = 8·10^−3^) and rs6850524 (p = 0.01, q = 0.02). However, we did not, find any significant association between rs13124436 polymorphism from CLOCK gene and male infertility. Under recessive genotype model, OR estimates ranged between 1.4 and 1.9 for CLOCK gene polymorphisms: rs11932595 (p = 6·10^−5^, OR = 1.9 with 95% CI 1.4–2.7), rs6811520 (p = 2·10^−3^, OR = 1.7 with 95% CI 1.2–2.2) and rs6850524 (p = 0.01, OR = 1.4 with 95% CI 1.1–1.9).

We also analyzed the inferred haplotypes in both investigated genes. The frequencies of predicted haplotypes in the study and the control group are presented in Table 3.

**Table 3:** Frequencies and distribution of probable haplotypes in the patient and control groups as predicted by haplo.stats.

	CLOCK gene							
	rs6811520	rs6850524	rs11932595	rs13124436	Frequency(patients)	Frequency(controls)	p	Simulated p value
**1**	T	C	A	G	0.21	0.28	8·10^−5^	5·10^−5^
**2**	C	G	A	G	0.05	0.06	0.49	0.48
**3**	C	G	G	A	0.09	0.09	0.20	0.20
**4**	C	G	A	A	0.13	0.10	0.14	0.14
**5**	C	G	G	G	0.20	0.17	0.04	0.04
**6**	C	C	G	G	0.07	0.04	8·10^−3^	8·10^−3^
	**ARNTL gene**							
	**rs3789327**	**rs1481892**	**rs4757144**	**rs12363415**	**Frequency** **(patients)**	**Frequency** **(controls)**	**p**	**Simulated p value**
**1**	T	G	G	A	0.11	0.12	0.28	0.28
**2**	T	G	A	A	0.21	0.22	0.62	0.62
**3**	T	C	G	A	0.10	0.11	0.73	0.72
**4**	C	G	G	A	0.08	0.08	0.69	0.70
**5**	C	G	A	A	0.15	0.12	0.07	0.07
**6**	C	C	G	A	0.06	0.04	0.07	0.06

A statistically significant difference in haplotype distributions was also confirmed at the CLOCK gene locus when comparing the frequencies of haplotypes TCAG (p = 8·10^−5^, simulated p value after 10.000 permutations was 5·10^−5^), CCGG (p = 8·10^−3^, simulated p value was 8·10^−3^) and CGGG (p = 0.04, simulated p value equaled to 0.04) between the infertile patients and fertile controls.

The SNPs interrogated in the ARNTL gene, rs3789327, rs1481892 and rs4757144 did not show significant associations of genotype or allelic distribution between the two groups.

Accordingly, we did not find any significant difference comparing the frequencies of 6 most frequent haplotypes for the 4 analyzed SNPs in the ARNTL gene in the study and control groups.

Power analyses were performed to estimate the lower sensitivity threshold of our study to detect the variants characterized by low-to-modest effect sizes. For this purpose, the pbsize2 function of gap package for R was utilized. Calculations showed that our power to detect a significant result in the presence of the actual genotype-phenotype effect with genotype relative risk equal to at least 1.7, was 82.3%, when taking into account the sample size, the significance threshold of 0.05, the prevalence of male infertility in the general population equal to 4.5%, and disease allele frequency of at least 10%, and considering multiplicative model of genetic association.

## Discussion

In the case-control study we found evidence of an association between male infertility and gene variants of the CLOCK gene in a sample of 961 men.

The circadian clock is internal timing system which allows an organism to provide environmental changes and adapt to them. Therefore, circadian rhythms manage a large variety of physiological and metabolic functions; any disruption of these rhythms may affect human health. Only a few studies have investigated the role of the circadian system in male fertility. It has been shown that night shift workers have an increased risk of infertility [16] and that infertile night shift workers have increased serotonin levels and decreased sperm quality compared to fertile night shift workers [17]. Serotonin is namely another component of the circadian system, potentially playing a role in human fertility [18] and is necessary for the development of normal spermatogenesis in rats [19]. More data is available regarding the role of the circadian system in male infertility in animals. A mutation of circadian genes influences reproductive fitness in Drosophila melanogaster. Beaver *et al*. (2002) have shown that clock-mutant males produce significantly fewer progeny, and release smaller quantities of sperm [20], whereas the studies of ARNTL knock-out mice have shown deficiencies in steroidogenesis [7], namely, male ARNTL KO mice had altered levels of reproductive hormones, indicating a defect in testicular Leydig cells. On the other hand, there is evidence that reproductive hormones directly influence the circadian system [21]; dysregulation of the either axis could therefore contribute to the reduced fertility. The circadian system influences testosterone production in humans, showing morning peaks and evening decrease [8], [9], [10]. Moreover, serum levels of sex steroid hormones have been associated with genetic variants in the circadian rhythm genes [11].

In women, genetic variability in the circadian rhythm genes ARNTL and NPAS2 has been suggested to contribute to fertility and seasonality, whereas the genetic variability in the ARNTL gene has been related to a higher number of pregnancies and also to a higher number of miscarriages; polymorphisms in the Npas2 gene have been associated with a decreased number of miscarriages [22].

In addition to its direct effects, the indirect effect of the circadian system on many physiological processes might possibly influence the male reproductive function. For example, the genetic variability in the CLOCK gene has been associated with increased weight and obesity [23], and loss of the ARNTL gene functions was shown to result in development of metabolic syndrome in knock-out rats [24]. Additionally, altered expression in the circadian rhythm genes has been shown to occur in obese males [25]. The negative effect of obesity on male reproductive function has been substantiated in numerous studies [26]–[28].

In conclusion, we provide evidence that genetic variability in the CLOCK gene might be associated with male infertility, and consequently imply the role of the circadian timing system in human reproduction. Further confirmation and mechanistic investigation of circadian system in male infertility is warranted.
